# Effects of *Helicobacter pylori* infection on T cell activation markers and regulatory T cells in people with and without HIV infection in Central Ethiopia

**DOI:** 10.1186/s40001-026-04025-4

**Published:** 2026-02-11

**Authors:** Smaranda Gliga, Million Getachew Mesfun, Tafese Beyene Tufa, Andre Fuchs, Hans Martin Orth, Edmund Osei Kuffour, Philipp A. Lang, Tom Luedde, Torsten Feldt

**Affiliations:** 1 Department of Gastroenterology, Hepatology and Infectious Diseases, University Hospital Düsseldorf, Heinrich Heine University, Düsseldorf, Germany; 2 Hirsch Institute of Tropical Medicine, Asella, Ethiopia; 3 Arsi University College of Health Sciences, Asella, Ethiopia; 4 Internal Medicine III – Gastroenterology and Infectious Diseases, University Hospital of Augsburg, Augsburg, Germany; 5 Institute of Molecular Medicine II, University Hospital Düsseldorf, Heinrich Heine University, Düsseldorf, Germany

**Keywords:** T cell exhaustion, T cell proliferation, *Helicobacter pylori* eradication therapy, Immune modulation, HIV

## Abstract

**Background:**

*Helicobacter pylori (H. pylori)* is known to modulate host immunity and sustain chronic inflammation, yet most data come from HIV-negative populations. In people living with HIV, whose T cell compartments are already dysregulated, the way *H. pylori* shapes peripheral T cell phenotypes, and how those profiles change after eradication therapy, is still unclear. Because both infections are common in Central Ethiopia, we examined peripheral T cell phenotypes in adults with and without HIV according to *H. pylori* status and assessed the immunologic effects of antibiotic eradication.

**Materials and methods:**

We conducted a prospective study in people with and without HIV infection from Ethiopia. *H. pylori* status was determined by stool-antigen testing; a subset received standard triple therapy and was followed for 12 months. Multiparameter flow cytometry quantified T cell activation, proliferation, exhaustion, and regulatory T cells (T_regs_) at baseline and after therapy.

**Results:**

T cell analyses showed that participants with HIV had consistently higher proliferation (Ki67), exhaustion (PD‐1, TIM3), and Th17 (CCR6⁺CD161⁺) markers than those without HIV. *H. pylori*-positive individuals exhibited higher T_reg_ levels irrespective of HIV status (HIV-negative: median 2% vs 1.08%, *p* < 0.0001; HIV-positive: median 2.9% vs 1.62%, *p* = 0.009). Successful eradication therapy led to a significant reduction in T_regs_ in both HIV-positive (median 3.04% → 0.70%, *p* = 0.031) and HIV-negative (median 2.96% → 1.46%, *p* = 0.040) groups. A similar decline was also observed in HIV-negative individuals with unsuccessful therapy (median 2.85% vs 1.29%, *p* = 0.0039).

**Conclusions:**

*H. pylori* infection was linked to significant differences in T cell profiles in both HIV-negative and HIV-positive individuals. Eradication therapy was followed by a reduction in Tregs—significant in HIV-negative participants irrespective of outcome and in PLWH with successful eradication—with subgroup-specific shifts in activation and differentiation/exhaustion markers, highlighting potential therapeutic avenues for mitigating immune dysregulation in co-infected populations.

**Supplementary Information:**

The online version contains supplementary material available at 10.1186/s40001-026-04025-4.

## Background

*Helicobacter pylori (H. pylori)* is a microaerophilic bacterium that establishes lifelong residence in the gastric epithelium after infection, which usually occurs in childhood [1]. Although most carriers remain symptom-free, a proportion progress to dyspepsia and other gastroduodenal disorders [2].

Early during colonization, innate sensing sparks the release of pro-inflammatory cytokines which in turn drive a strong T helper (Th) 1 and Th17 skewed T-cell response [3, 4]. To prevent excessive tissue damage, the mucosa simultaneously produces counter-regulatory mediators such as transforming growth factor (TGF)-β and IL-10 [5]. TGF-β recruits T cells with anti-inflammatory activity, in particular regulatory T cells (T_regs_). T_regs_ derive from the same lineage as naïve CD4^+^ T cells and present the transcription factor forkhead box protein 3 (Foxp3) and the activating marker CD25 [6]. It was shown that a greater population of T_regs_ is present in the gastric mucosa and in the peripheral blood of *H. pylori* positive individuals, while T_regs_ are largely absent in *H. pylori* negative subjects [7, 8]. Although T_regs_ are activated to prevent hyperinflammatory conditions, they can also facilitate bacterial persistence by suppressing IFN-γ-producing effector T cells [9]. Consistent with this notion, experimental depletion of T_regs_ with anti-CD25 antibodies in mice intensifies gastritis while reducing the gastric bacterial load, highlighting the delicate balance between protection and pathology [10].

Given that a significant proportion of the 39 million people living with HIV (PLWH) [11] worldwide reside in low- and middle-income countries, where *H. pylori* infection is highly prevalent, it is likely that many of these individuals also have *H. pylori* co-infection. HIV infection is characterized by a reduced CD4^+^ T cell count, which in time favors opportunistic infections, both viral and bacterial. Most studies report a lower prevalence of *H. pylori* infection among PLWH, particularly in those with advanced immunodeficiency (CD4⁺ T cells < 200/µL), as documented in cohorts from Brazil [12], Argentina [13], Ghana [14], and the United States [15]. By contrast, an earlier Ethiopian study found higher prevalence in PLWH compared to HIV-negative individuals [16], and a more recent investigation from Northern Ethiopia confirmed this trend, also identifying associated risk factors [17]. In Serbia, prevalence among PLWH rose during the HAART era to levels comparable with HIV-negative controls [18]. These discrepancies likely reflect a combination of geographical variation, immune status, ART-mediated immune reconstitution, and differences in diagnostic approaches.

Furthermore, the introduction of combination antiretroviral therapy (cART) induced an increase in dyspeptic complications and their severity [19]. HIV infection is associated with a strong recruitment of Th2 cells and reduction of Th1/Th17cells, which in turn decrease the pro-inflammatory response against *H. pylori* and the intensity of gastritis [20].

A previous study our group performed in Ghana showed that *H. pylori* carriage coincided with reduced CD4⁺ T cell activation (HLA-DR⁺CD38⁺), proliferation (Ki67) and PD-1 expression in both ART-naïve PLWH and HIV-negative controls [14]. These results parallel other reports in which co-infected, treatment-naïve patients displayed comparatively higher CD4⁺ counts and diminished HIV-1 viral loads, supporting the notion that the bacterium can dampen systemic immune activation [16, 18]. To date, however, no study has characterized these immunological interactions in Ethiopian cohorts, nor has any work prospectively examined how eliminating *H. pylori* affects T cell phenotypes in this setting. Accordingly, we designed the present prospective, randomized study to elucidate the mechanisms of systemic immune modulation linked to *H. pylori* infection by tracking activation, proliferation, and exhaustion signatures before and after standard eradication therapy.

## Materials and methods

### Study population

Between March and June 2017, we enrolled adults attending the Asella Teaching and Referral Hospital (ATRH, Ethiopia). Two streams were used: (i) successive visitors to the HIV outpatient service and (ii) successive clients of the hospital’s voluntary counselling-and-testing unit. All recruits underwent *H. pylori* screening with a stool-antigen assay (see below). Eligibility criteria were 18–55 years of age, signed informed consent and for PLWH, a CD4⁺ cell count above 350 cells/µl. From the first 140 HIV-negative and 140 HIV-positive individuals who satisfied these criteria we applied additional exclusion filters: prior immunosuppression, malignancy, chronic viral hepatitis (B and C), tuberculosis, elevated C-reactive protein, parasitic infection, recent anti-helminthic/antibiotic therapy, upper-gastrointestinal symptoms, recent acute infection, hemoglobin < 10 g/dl or pregnancy, as described earlier ^15^. All PLWH were on cART at study inclusion.

### Ethics

The protocol received approval in Ethiopia and Germany (refs A/U/H/S/C/87/6392; 3.10/271/2017; 5728).

### Eradication therapy

Within the *H. pylori*–positive group, 26/123 HIV-negative individuals and 25/121 PLWH were randomized to receive a 14-day triple regimen: metronidazole 500 mg, clarithromycin 500 mg and pantoprazole 40 mg, each taken twice daily. This macrolide- and nitroimidazole-based schedule follows Ethiopian guidelines and deliberately avoids penicillin to prevent reactions in participants with undocumented β-lactam allergy, as previously mentioned [21]. Clinical review with paired blood and stool sampling was scheduled at baseline and at three, 6 and 12 months. Successful eradication was defined as a negative stool-antigen test at the three-month visit.

### *Helicobacter pylori* stool antigen test

Antigen detection employed the GA Generic Assays kit (Blankenfelde, Germany) according to the manufacturer’s protocol. Stool microscopy with direct and formol-ether concentration techniques ruled out concomitant parasitic infection.

### Blood sample collection and laboratory analysis

At every visit 15 ml of EDTA-anticoagulated blood and 5 ml of serum were collected. Serum at baseline was analyzed for hepatitis B surface antigen (HBsAg), anti-hepatitis C (HCV) antibodies (InTec Products Inc., China) and CRP (NADAL^®^ CRP, nal von minden, Germany). Complete blood counts and, for PLWH, CD4⁺ T cell enumerations were performed locally on a FACSCalibur™ flow cytometer (BD Biosciences, NJ, USA).

### PBMC isolation and cryopreservation

Peripheral blood mononuclear cells (PBMCs) were isolated by Ficoll-Hypaque density gradient, suspended in 20% dimethyl sulfoxide (DMSO)/fetal calf serum (FCS) freezing medium, cooled at − 80 °C for 24 h in a controlled-rate container and shipped on dry ice to Düsseldorf for batched analysis.

### Flow-cytometric assessment

**Fig. 1:**
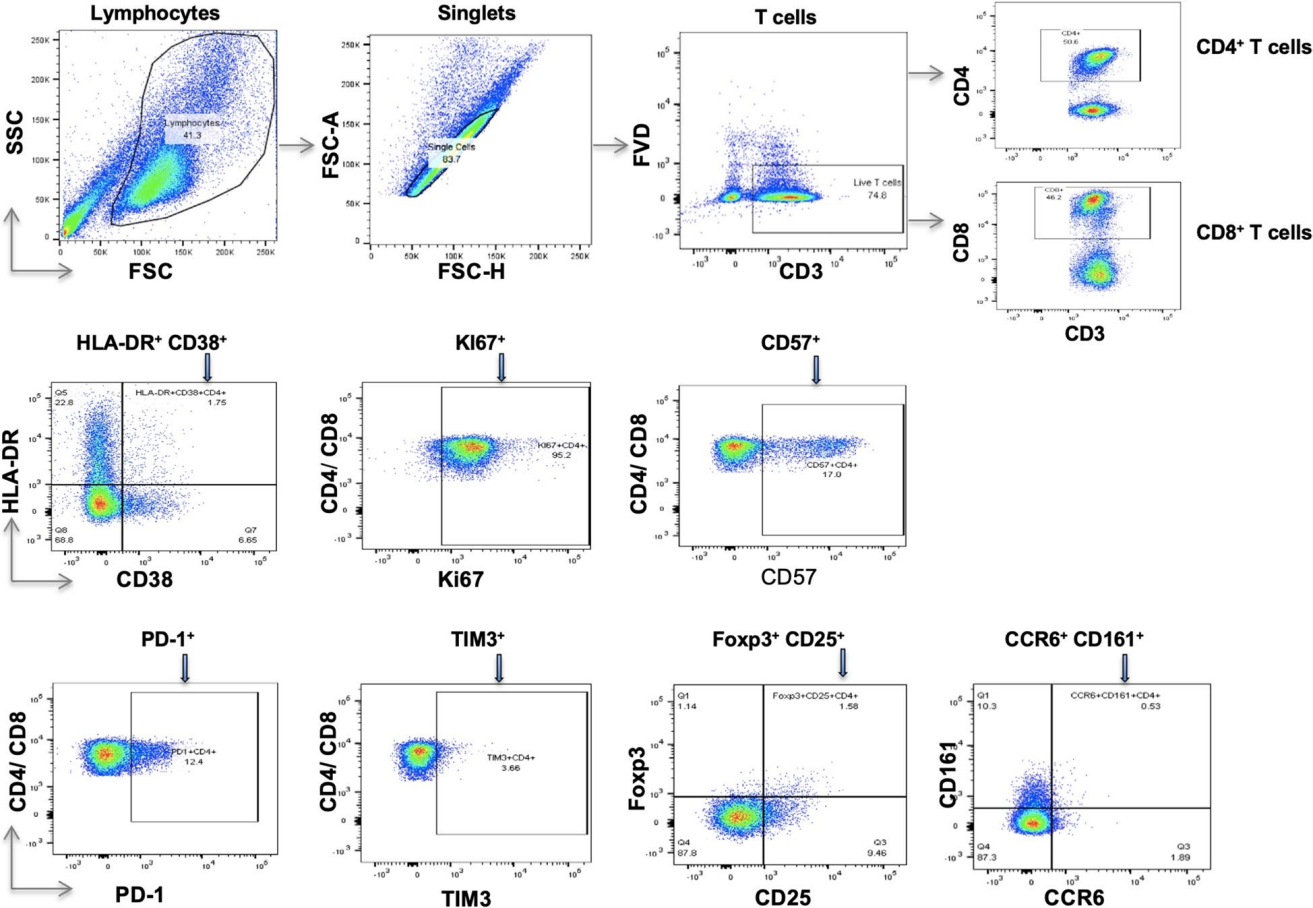
Gating of lymphocytes, singlets and T cell subpopulations. *FSC* forward scatter, *SSC* sideways scatter, *FSC-H* forward scatter height, *FSC-A* forward scatter area, *FVD* fixable viability dye

To evaluate how *H. pylori* clearance altered T cell characteristics, phenotypic read-outs recorded after therapy were compared with each participant’s own baseline. Cryopreserved PBMCs were rapidly thawed at 37 °C, washed twice and resuspended in phosphate-buffered saline (PBS). Surface labelling was performed at 2–8 °C in the dark, whereas intracellular markers were stained at room temperature. Antibody details are listed in Supplementary Table 1; four dedicated panels covered (1) activation, (2) exhaustion, (3) regulatory T cells and (4) Th17 signatures. All reagents were sourced from eBioscience (Frankfurt am Main, Germany). Viable cells were gated with a fixable viability dye. After surface staining, samples in panels 2 and 4 were held in flow-cytometry buffer for immediate acquisition, while those in panels 1 and 3 underwent fixation, permeabilization and 30-min intracellular staining before a final wash into buffer. Data were collected on a three-laser BD FACSCanto II. Spectral overlap was corrected with UltraComp beads (eBioscience, Germany), singularly stained for each fluorochrome. Files were processed in FlowJo v10.1 (Tree Star, OR, USA); the gating strategy appears in Fig. 1. Phenotypic outputs were stratified by HIV status and by *H. pylori* infection, as well as by pre- versus post-eradication time points.

### Statistical analysis

Data from participants without and with HIV were processed independently with Prism version 9.0 (GraphPad Software, San Diego, California, USA) software. Group comparisons used two-tailed Mann–Whitney U tests (unpaired) and Wilcoxon signed-rank tests (paired). Raw p-values were used for the primary analyses and are displayed in all figures. Results are reported as medians with 95% confidence intervals (CI) for unpaired comparisons and as medians for paired comparisons. To address multiplicity within each marker family, we report Bonferroni thresholds (applied separately for CD4⁺ and CD8⁺ panels) and explicitly indicate in the text which findings meet these corrected cut-offs. Significance codes: *n.s*. = not significant; *p < 0.05; **p < 0.01; ***p < 0.001; ****p < 0.0001, unless stated otherwise in the figure and text.

## Results

### Cohort

**Fig. 2:**
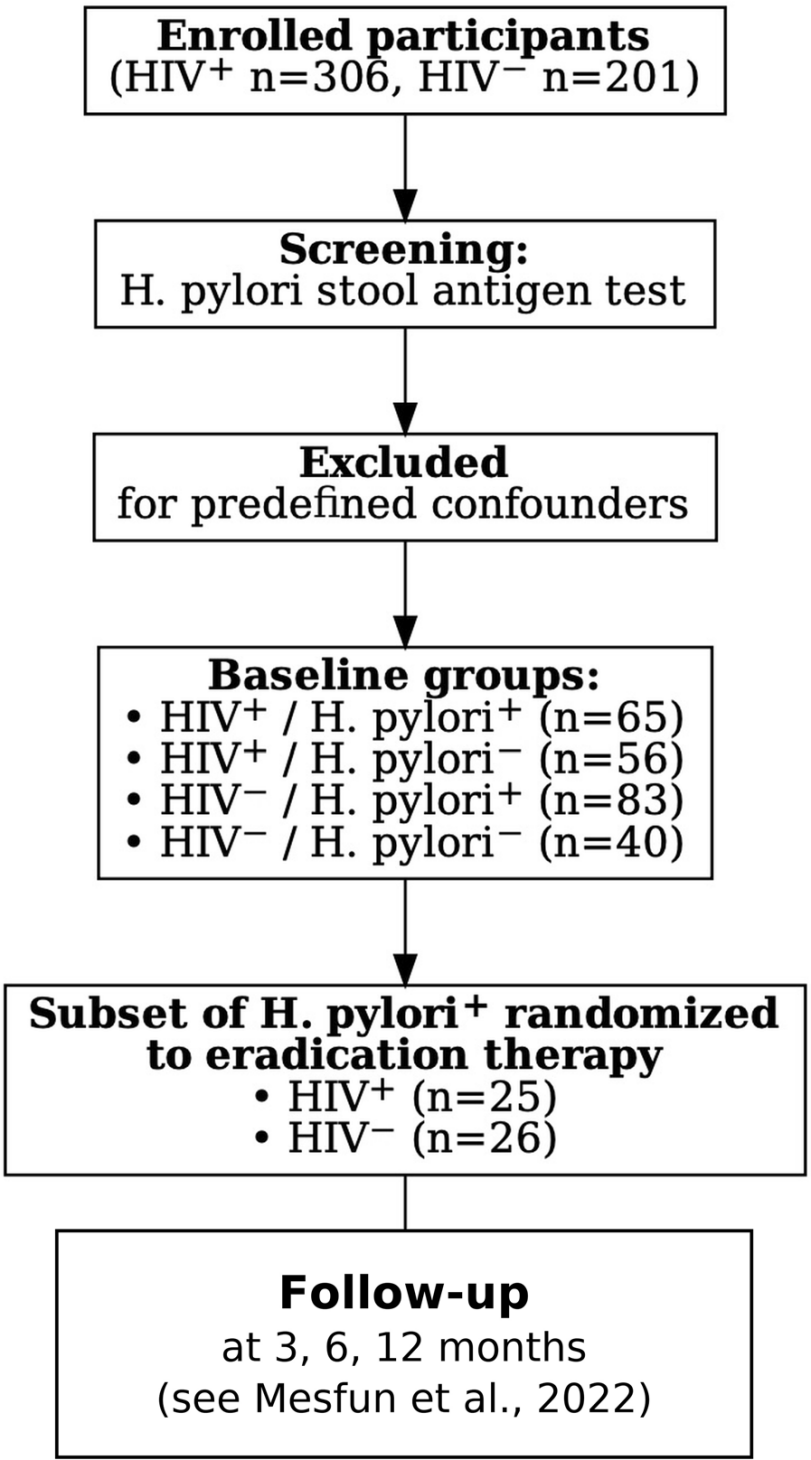
Simplified study flow and group allocation. A total of 306 HIV⁺ and 201 HIV⁻ individuals were screened for *H. pylori* infection by stool antigen testing. After exclusion of participants with predefined confounders, four baseline groups were established: HIV⁺/*H. pylori*⁺ (*n* = 65), HIV⁺/*H. pylori*⁻ (*n* = 56), HIV⁻/*H. pylori*⁺ (*n* = 83), and HIV⁻/*H. pylori*⁻ (*n* = 40). From the *H. pylori*⁺ cohorts, 25 HIV⁺ and 26 HIV⁻ participants were randomized to a 14-day eradication therapy regimen. Follow-up assessments were scheduled at 3, 6, and 12 months. Detailed participant flow and attrition are available in Mesfun et al. [21]

A cohort of 306 people living with HIV and 201 people without HIV underwent screening to detect *H. pylori* colonization at enrolment. From these, we pragmatically selected 140 individuals in the HIV-positive cohort (78 colonized, 62 non-colonized) and an equal 140 individuals in the HIV-negative cohort (93 colonized, 47 non-colonized) for detailed evaluation of exclusion criteria, including co-infections, inflammation, medication, or pregnancy history. Subsequently, 27 *H. pylori* positive volunteers from each cohort were randomly allocated to a 14-day metronidazole–clarithromycin–pantoprazole regimen, constituting the study’s standard triple eradication intervention arm for bacterial clearance. The overall study design and group allocation are summarized in Fig. 2. After testing for confounding factors (specified above), two participants from the group with HIV and one participant from those without HIV infection were excluded from analysis as predefined. The rates of successful eradication in participants with HIV and those without HIV were previously reported [21].

### Comparison of T cell profile in participants without vs. with HIV at baseline, irrespective of *H. pylori* status

**Fig. 3:**
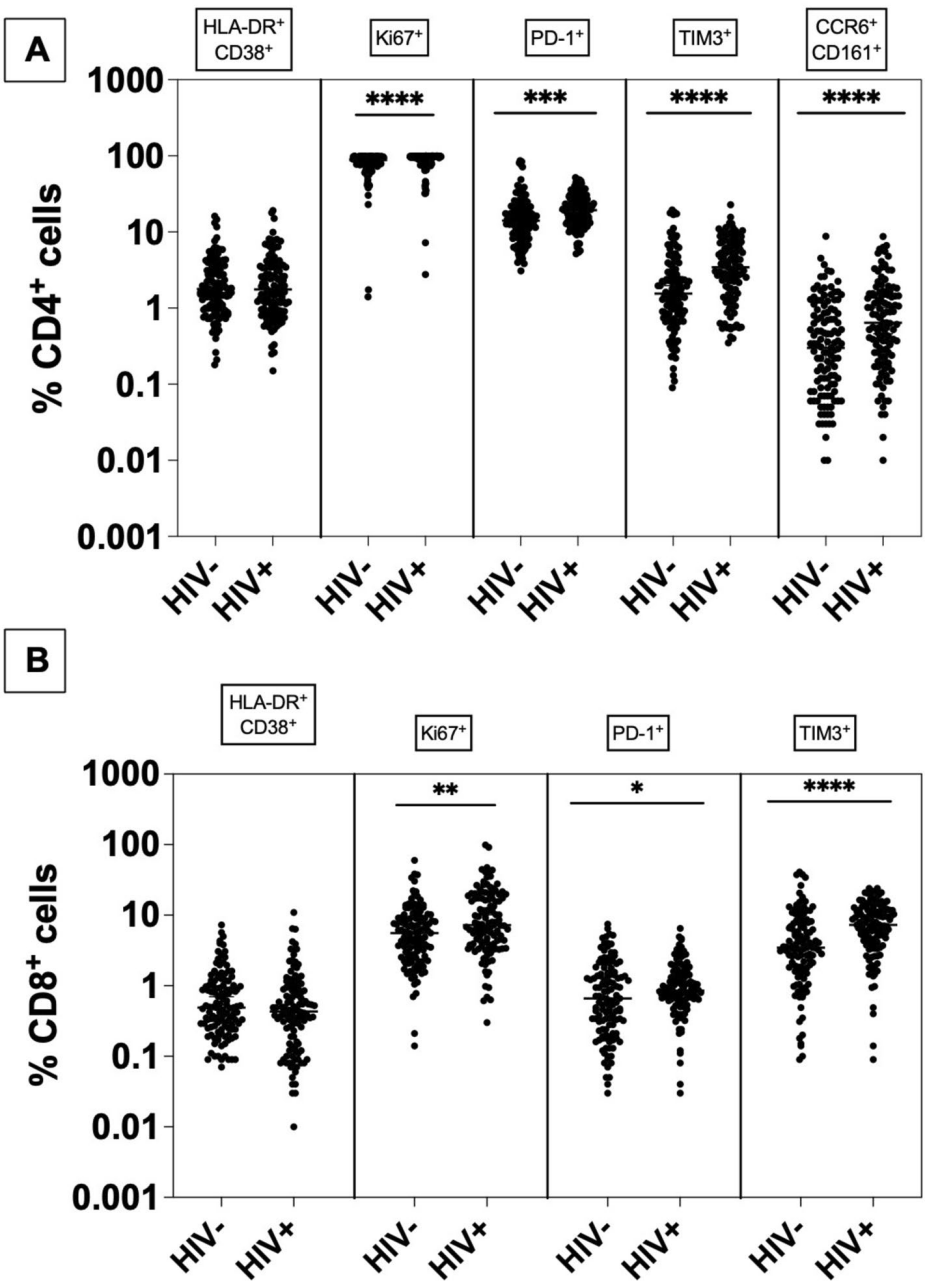
T cell activation, proliferation, exhaustion, and Th17 markers in HIV-negative versus HIV-positive participants. **A** CD4⁺ T cells: expression of HLA-DR⁺CD38⁺, Ki67, PD-1, TIM-3, and Th17 (CCR6⁺CD161⁺). **B** CD8⁺ T cells: expression of HLA-DR⁺CD38⁺, Ki67, PD-1, and TIM-3. Each dot represents one individual; horizontal bars indicate the median with 95% CI. Group comparisons were performed using the two-tailed Mann–Whitney *U* test. *P*-values were adjusted for multiple comparisons using the Bonferroni method (five markers for CD4⁺—*α* = 0.01, four markers for CD8⁺—α = 0.0125). Sample sizes: HIV^−^ *n* = 123; HIV^+^ *n* = 121. For the *y* axis a logarithmic scale was used. *n.s*. not significant and *p < 0.05, **p < 0.01, *** *p* < 0.001 and **** *p* < 0.0001

Irrespective of *H. pylori* status, CD4⁺ T cells from participants with HIV exhibited consistently higher levels of Ki67 (median 96% vs 88%; Mann–Whitney *p* < 0.0001; Bonferroni-adjusted *p* < 0.0001) as a proliferation marker, as well as elevated PD-1 (median 19% vs 14%; Mann–Whitney *p* < 0.001; Bonferroni-adjusted *p* < 0.01) and TIM-3 expression (median 3.4% vs 1.6%; Mann–Whitney *p* < 0.0001; Bonferroni-adjusted *p* < 0.0001) as markers of exhaustion. Th17 cells (CCR6⁺CD161⁺) were also increased (median 0.64% vs 0.30%; Mann–Whitney *p* < 0.0001; Bonferroni-adjusted *p* < 0.001). No significant difference was observed for HLA-DR⁺CD38⁺ expression (Mann–Whitney *p* = 0.91; Bonferroni-adjusted *p* = 1.0) (Fig. 3A).

In addition, CD8⁺ T cells displayed higher levels of Ki67 (median 7.2% vs 5.6%; Mann–Whitney *p* = 0.0010; Bonferroni-adjusted *p* = 0.0041) and TIM-3 (median 7.2% vs 3.4%; Mann–Whitney *p *< 0.0001; Bonferroni-adjusted *p* < 0.0001), whereas PD-1 showed only a modest increase (median 0.84% vs 0.66%; Mann–Whitney *p* = 0.039; Bonferroni-adjusted *p* = 0.16) and HLA-DR⁺CD38⁺ did not differ significantly (*p* = 0.10; Bonferroni-adjusted *p* = 0.41) (Fig. 3B). All values, including medians with 95% confidence intervals, are summarized in Supplementary Table 2.

### T cell profile in participants without HIV according to *H. pylori* status at baseline

**Fig. 4:**
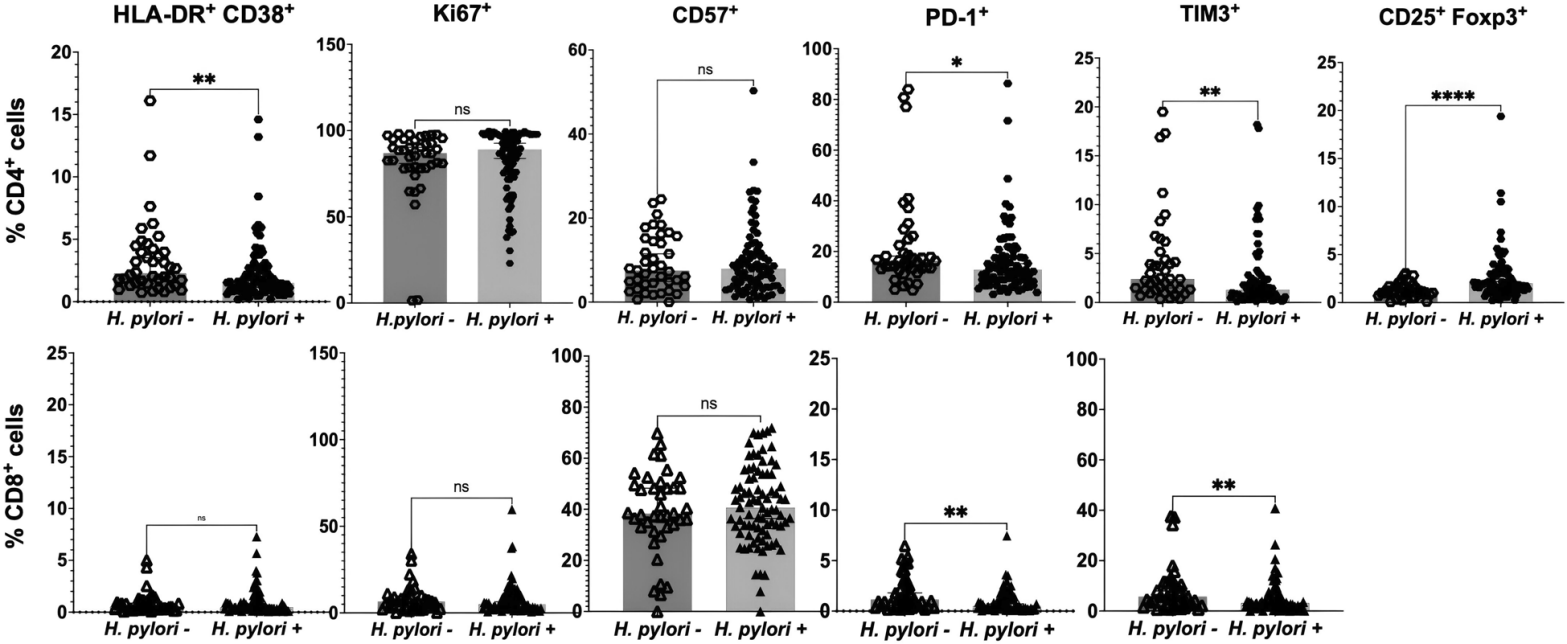
Peripheral T cell parameters compared between *H. pylori*–uninfected and infected individuals without HIV. Scatter plots display medians with 95% confidence intervals (CI). CD4⁺ data (upper row, circles; *H. pylori*⁻ *n* = 40, *H. pylori*⁺ *n* = 83) and CD8⁺ data (lower row, triangles; *H. pylori*⁻ *n* = 40, *H. pylori*⁺ *n* = 83) are shown. Statistical evaluation employed the Mann–Whitney *U* test. *p*-values shown are raw values; Bonferroni correction was applied manually, separately for CD4⁺ (*m* = 7, *α* = 0.0071) and CD8⁺ (*m* = 4, *α* = 0.0125) subsets. Markers that remained significant after correction are noted in the text. n.s. denotes non-significant; *p < 0.05, **p < 0.01, ****p < 0.0001

In the group of participants without HIV (*n* = 123 after exclusion of 17 participants with predefined confounding factors such as co-infections, inflammation, or pregnancy; see Methods for details), T cell markers were analyzed comparatively between subgroups with (*n* = 83) and without (*n* = 40) *H. pylori* infection. Results for CD4^+^ and CD8^+^ T cells are presented in Fig. 4.

In HIV-negative participants, *H. pylori*⁻ individuals had higher frequencies of CD4⁺ T cells expressing HLA-DR⁺CD38⁺ compared to *H. pylori*⁺ (median 2.24% vs 1.52%; *p* = 0.003). Similarly, exhaustion markers were more frequent in *H. pylori*⁻ compared to *H. pylori*⁺ individuals: PD-1 on CD4⁺ (median 16.05% vs 12.7%; *p* = 0.030) and CD8⁺ T cells (median 1.12% vs 0.49%; *p* = 0.001), as well as TIM-3 on CD4⁺ (median 2.39% vs 1.28%; *p* = 0.002) and CD8⁺ T cells (median 5.61% vs 3.13%; *p* = 0.007). In contrast, T_regs_ (CD25⁺Foxp3⁺CD4⁺) were more frequent in *H. pylori*⁺ compared with *H. pylori*⁻ individuals (median 2.0% vs 1.08%; *p* < 0.0001). After Bonferroni correction for multiple comparisons (CD4: *m* = 7, *α* = 0.0071; CD8: *m* = 4, *α* = 0.0125), the following remained statistically significant: higher CD4⁺ HLA-DR⁺CD38⁺ and lower CD4⁺ TIM-3 expression, higher Treg frequencies in H. pylori⁺ compared to H. pylori⁻, and lower CD8⁺ PD-1 and TIM-3 expression in H. pylori⁺ individuals. Other differences were nominally significant but did not pass the adjusted thresholds.

### T cell profile in participants with HIV infection according to *H. pylori* status at baseline

**Fig. 5:**
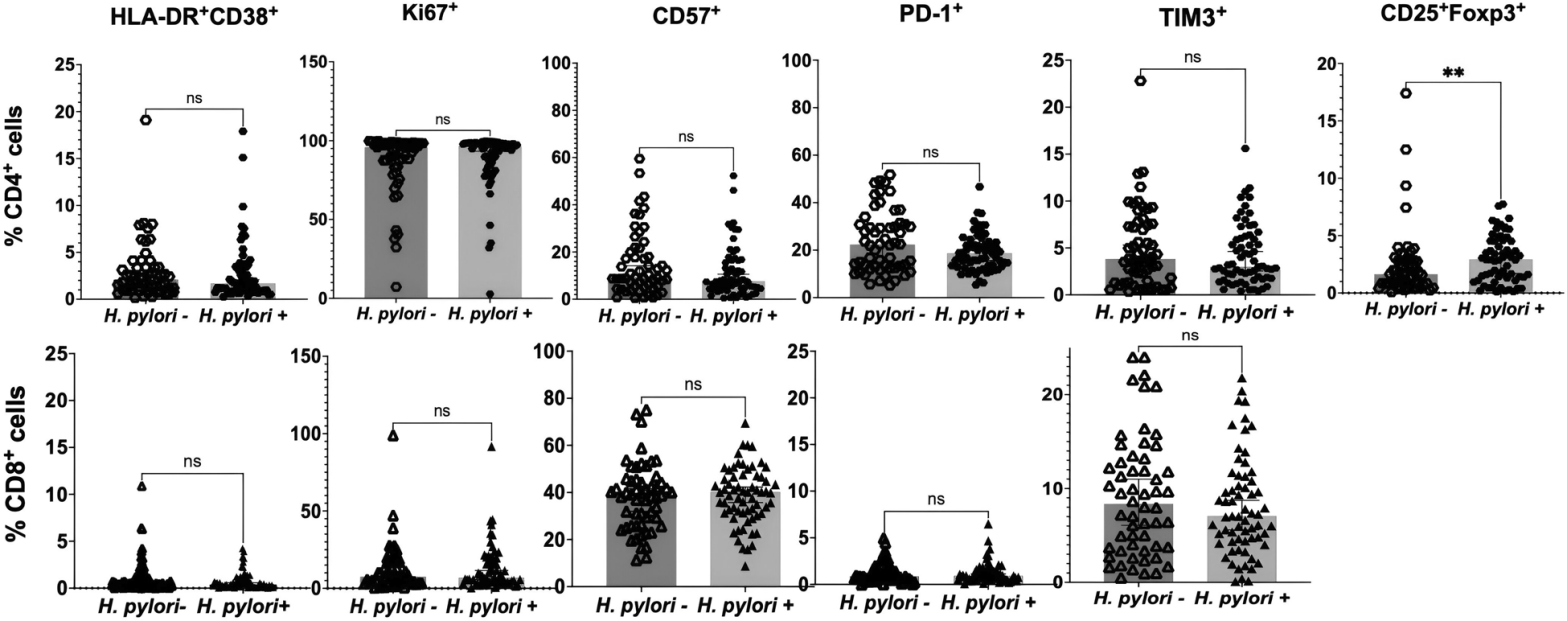
Peripheral T cell parameters compared between *H. pylori*^−^ (*n *= 56) and *H. pylori*^+^ (*n* = 65) individuals in people living with HIV (PLWH). Statistical evaluation employed the Mann–Whitney *U* test; raw *p*-values are shown in the plots. Graphs display medians with 95% confidence intervals (CI) CD4⁺ data (upper row) use polygon symbols, whereas CD8⁺ data (lower row) use triangles throughout all scatter plots. n.s. denotes non-significant; **p < 0.01. For multiple testing, Bonferroni correction was applied (*m* = 7 for CD4⁺, *α* = 0.0071; *m* = 4 for CD8⁺, *α* = 0.0125), and results that remained significant are reported in the text

Among PLWH (*n* = 121), *H. pylori*^−^ individuals (*n* = 56; left in Fig. 5) were compared with *H. pylori*^+^ individuals (*n* = 65; right). Regulatory T cells (CD25⁺Foxp3⁺CD4⁺) were higher in the *H. pylori*^+^ subgroup (median 1.62% vs 2.90%; Mann–Whitney *p* = 0.009). After Bonferroni correction for multiple CD4⁺ comparisons **(m** = 7; *α* = 0.0071), this difference no longer reached statistical significance (adjusted *p* = 0.063).

No significant differences in Th17 cell frequencies (CCR6⁺CD161⁺CD4⁺) were observed between *H. pylori*⁺ and *H. pylori*⁻ subgroups, irrespective of HIV status (Supplementary Fig. 1).

### Effect of *H. pylori* eradication therapy on T cell populations

At the 3-month follow-up, successful eradication, confirmed by a negative H. pylori stool antigen test, was documented in 15/24 HIV-negative participants (62.5%) and 9/24 participants with HIV (37.5%). At the second follow-up (3 months later, i.e. between 3 and 6 months after eradication therapy), two individuals in the HIV group and one in the HIV-negative group showed recurrence/reinfection and were excluded. This left a total of 7 participants with HIV and 14 without HIV eligible for the final analysis (for details see Mesfun et al.) [21].

**Fig. 6:**
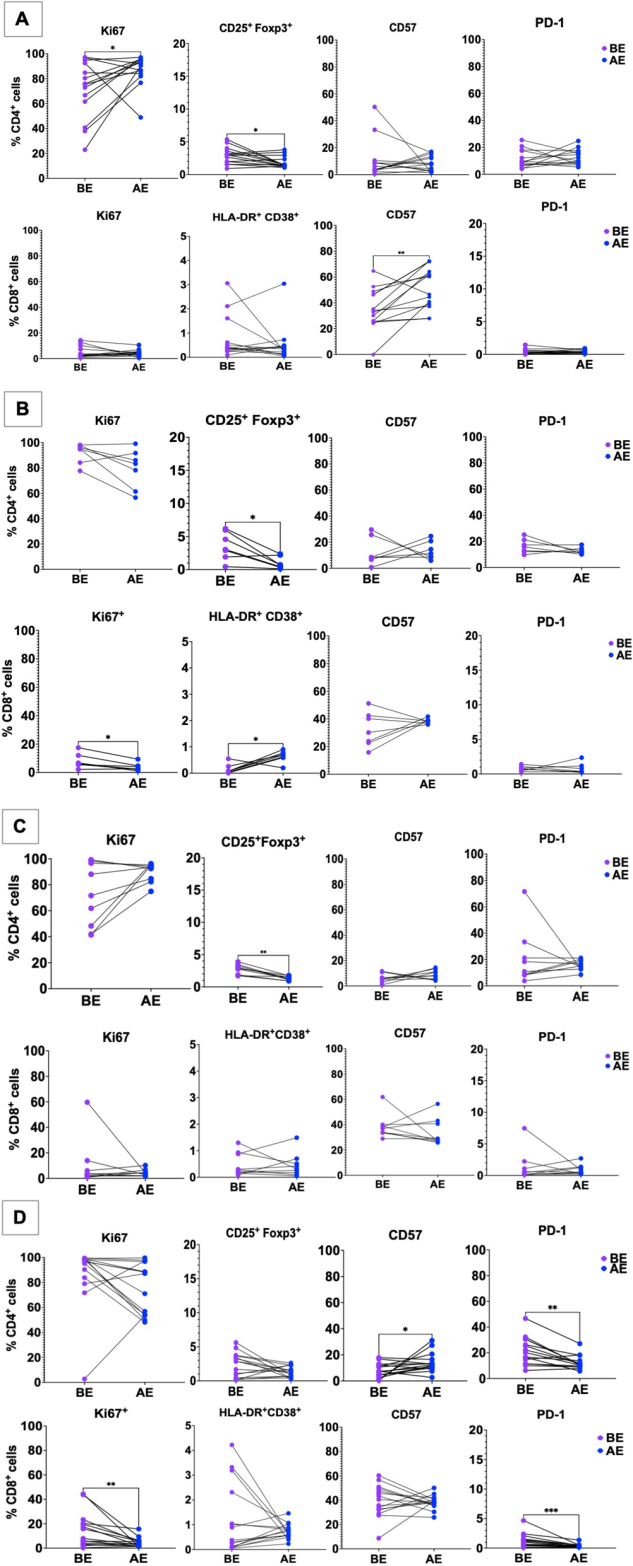
T cell profile before (BE) and after (AE) eradication therapy. Paired comparisons before (BE) and after eradication therapy (AE) are shown for HIV-negative individuals with successful eradication (**A**; *n* = 14), PLWH with successful eradication (**B**; *n* = 7), HIV-negative individuals with failed eradication (**C**; *n* = 9), and PLWH with failed eradication (**D**; *n* = 15). Here, AE refers to the time point after completion of eradication therapy, irrespective of treatment success. Each point represents one patient; lines connect paired samples. Statistical evaluation was performed using the paired Wilcoxon signed-rank test. Raw p-values are shown in the figure. Multiple testing was corrected using the Bonferroni method (*α* = 0.0125 per subgroup). *p < 0.05, **p < 0.01, ***p < 0.001

In participants without HIV infection, successful *H. pylori* eradication resulted in increased proliferation marker expression (Ki67) in CD4^+^ T cells (*n* = 14; median 75.7 vs 91.3%, *p* = 0.049). CD8^+^ T cells showed an increase in exhaustion markers (CD57) (median 33.6% vs 53.4%, *p* = 0.0031; Fig. 6A). We observed a decrease in T_regs_ in HIV-negative participants after successful (median 2.96% vs 1.46%, *p* = 0.04), but also after unsuccessful (median 2.85% vs 1.29%, *p* = 0.0039; Fig. 6C) *H. pylori* eradication therapy. After Bonferroni correction (*α* = 0.0125), only the increase in CD8⁺ CD57⁺ T cells and the decrease in CD25⁺Foxp3⁺ Tregs after failed eradication remained significant, while all other changes lost significance under the adjusted threshold.

Among PLWH, seven individuals who cleared *H. pylori* showed a rise in CD8⁺ T cell activation (HLA-DR⁺CD38⁺; median 0.08% vs 0.69%, *p* = 0.031) accompanied by a fall in Ki67 proliferation signals (median 6.05% vs 2.64%, *p* = 0.031; Fig. 6B) and a parallel contraction of T_regs_ (median 3.04% vs 0.7%, *p* = 0.031). Conversely, in those whose therapy failed we detected reduced Ki67 on CD8⁺ T lymphocytes (median 7.9 vs 3.34%, *p* = 0.0026), lower PD-1 on CD4⁺ (median 20.2% vs 11.1%, *p* = 0.0015) and CD8⁺ T cells (median 0.99 vs 0.42%, *p* = 0.0009), together with elevated CD57 on CD4⁺ T cells (median 7.28 vs 12.5%, *p* = 0.03; Fig. 6D). Paired analyses of T_regs_ showed no significant change in this subgroup. After Bonferroni correction (*α* = 0.0125), only CD8⁺ Ki67, CD4⁺ PD-1, and CD8⁺ PD-1 remained significant.These shifts collectively suggest differential immune recalibration following attempted bacterial clearance in adults.

## Discussion

Because few studies from sub-Saharan Africa have explored how *H. pylori* shapes immune responses, this study aimed to fill this critical gap by prospectively examining *H. pylori*–related effects on T cell populations, including markers of activation, exhaustion and T_regs_, in individuals with and without HIV infection. The rationale for such an investigation stems from the potential immune‐modulating effects of *H. pylori*, which may be especially pertinent in the setting of HIV, where the immune system is already under strain.

Our study revealed that T cell exhaustion markers were consistently elevated in individuals with HIV, in line with well-established data on PD-1–mediated immune dysregulation [22, 23]. In addition, we observed higher TIM-3 expression on both CD4⁺ and CD8⁺ T cells, consistent with recent reports identifying TIM-3 as a marker of advanced or persistent T cell dysfunction in HIV infection, including under ART [24, 25].

Moreover, despite limited regional data on *H. pylori* immune interactions in Sub-Saharan Africa, we confirmed that in this cohort, HIV infection correlated with characteristic shifts in T cell activation, proliferative activity, and the distribution of regulatory subsets.

Building on these findings, we also evaluated Th17 cells, which tend to be depleted in untreated HIV but can be reconstituted under cART. Here, we noted persistently elevated Th17 markers in the HIV positive group, likely reflecting the relatively preserved CD4⁺ T cell counts afforded by ART. This backdrop provides important context for interpreting the role of *H. pylori*, a bacterium often linked to robust Th17 responses in gastric lesions [26–28]. In our cohort, however, *H. pylori* status did not appear to further alter Th17 frequency in people with HIV, indicating that cART-driven immune restoration may outweigh any additive influence from *H. pylori*. Studies examining *H. pylori*-driven changes in peripheral T cell activation, proliferation, and exhaustion remain limited. In our earlier work among ART-naïve individuals in Ghana, colonization corresponded to diminished CD4⁺ T cell activation (HLA-DR⁺CD38⁺) and lower expression of exhaustion markers, PD-1 on CD8⁺ cells and TIM-3 on both CD4⁺ and CD8⁺ subsets [14]. By contrast, in our current cART-treated cohort, we did not observe the same reduction in exhaustion markers, suggesting that effective HIV therapy may modify how *H. pylori* shapes systemic T cell phenotypes. A different group, however, documented increased HLA‐DR expression in PBMCs, especially in individuals with peptic ulcers, implying that *H. pylori* could have variable immunomodulatory effects depending on clinical presentation [29]. Other contrasting results come from murine and human studies [30, 31] describing elevated TIM3 expression in Th cells from *H. pylori*‐infected hosts, and higher exhaustion levels on CD8⁺ T cells in gastric cancer patients [31]. Such inconsistencies may reflect differences in host genetics, regional factors, sample size or disease phenotypes (e.g., asymptomatic carriers vs. peptic ulcer disease vs. gastric cancer). Our exclusive enrollment of asymptomatic patients could partly explain why we observed lower levels of certain exhaustion or activation markers relative to other findings.

A significant outcome of our investigation was the notably higher percentage of T_regs_ among *H. pylori*‐infected participants, regardless of HIV status, paralleling observations from histopathological studies of the gastric mucosa [7–9, 26, 32]. While most prior publications focused on local T_regs_ in gastric tissue, only two reports have shown elevated circulating T_regs_ in *H. pylori*‐infected cohorts [8, 26]. Our own earlier work hinted at a similar peripheral trend, suggesting that *H. pylori*–induced immune regulation may be systemic rather than confined solely to the gastric microenvironment [14].

Prior work by Eberhardt and colleagues [14] linked *H. pylori* infection to reduced T cell activation, proliferation and exhaustion markers, but could not definitively establish causality. Accordingly, we sought to determine whether eradicating *H. pylori* would lead to an immunological reversal of these trends. However, high levels of metronidazole resistance [33] in Ethiopia resulted in a low overall eradication rate, leaving only small subgroups available for a detailed pre‐ vs. post‐therapy comparison. Recognizing that *H. pylori* typically recruits T_regs_ to facilitate its persistence [32], we hypothesized that T_reg_ levels would drop if *H. pylori* were successfully eliminated. Indeed, we observed a decline in circulating Tregs after successful eradication in PLWH and after both successful and unsuccessful therapy in HIV-negative individuals. The latter suggests antibiotic- or microbiome-mediated effects independent of bacterial clearance [34, 35]. This broader disruption of the microbiota may have secondary effects on T_regs_ and Th17 subsets, mirroring data from other pathologies where antibiotic therapy modifies immune populations [36].

Interestingly, participants with HIV who were successfully eradicated showed increased activation of CD8⁺ T cells. We surmise that reduced T_reg_‐mediated suppression could permit cytotoxic T lymphocytes to become more activated [37]. Unexpectedly, those who failed eradication still exhibited a decrease in T_regs_ alongside reduced proliferation and PD‐1 expression, hinting at a complex interplay between T_regs_ and PD‐1/PD‐L1 pathways [38]. These findings complicate a straightforward narrative of T_reg_ modulation, pointing to additional factors, such as partial antibiotic effects, subclinical shifts in gut flora or baseline immune status, that may influence immune cell phenotypes regardless of eradication outcome.

Because multiple predefined T cell markers were analyzed in parallel, we applied Bonferroni correction within each marker family. While effect directions were consistent, only a subset surpassed the corrected thresholds: higher circulating Tregs and lower CD8⁺ PD-1 at baseline (between-group analyses), increased CD8⁺ CD57 in HIV-negative individuals with successful eradication, reduced T_regs_ in those with eradication failure, and decreases in Ki67 (CD8⁺) and PD-1 (CD4⁺, CD8⁺) among PLWH with failed eradication. Given the conservatism of Bonferroni, trends not meeting the adjusted α may still be biologically meaningful and warrant confirmation in larger cohorts.

Although we document significant immunological shifts following *H. pylori* eradication, the limited sample size and suboptimal eradication success rate constrain our ability to draw broad conclusions. Larger-scale studies using alternative antibiotic regimens or susceptibility-guided therapy are needed to confirm these findings and clarify the complex interplay among *H. pylori*, HIV status, antibiotic resistance and host genetics. Furthermore, we lacked contemporaneous HIV viral-load measurements, preventing correlation of immune changes with virologic control.

Moreover, while antibiotics may affect T_regs_ and Th17 cells, we did not systematically evaluate broader gut microbiome shifts, which can transiently or persistently alter immune phenotypes and confound outcomes [39, 40]. Future investigations should integrate microbiome analyses (e.g., 16S rRNA or metagenomic approaches) to distinguish the effects of *H. pylori* eradication from those of collateral disruptions in gut flora.

Finally, despite observing immunological changes associated with *H. pylori* infection, the long-term consequences for HIV progression remain unclear. Some research suggests that chronic coinfections can modulate systemic immune activation in people with HIV, possibly influencing viral reservoirs or disease progression [41, 42]. Whether *H. pylori* is ultimately protective, neutral or detrimental may depend on bacterial strain diversity, host genetics, cART status, and local factors. Further exploration of these variables is needed to unravel the multifaceted relationship between *H. pylori* and HIV.

## Conclusions

This study underscores the importance of T_regs_ in modulating immune responses during *H. pylori* infection, regardless of HIV status. T_reg_ levels were consistently elevated in infected individuals, potentially aiding bacterial persistence, and successful *H. pylori* eradication led to a significant drop in T_regs_ with concurrent subgroup-specific shifts in activation and differentiation/exhaustion markers. Although these findings suggest potential therapeutic benefits by modulating T_regs_, the low eradication rate and limited sample size limit broader conclusions. Larger studies are needed to confirm these results, clarify clinical applications, and investigate whether targeting T_regs_ could benefit individuals with coinfections. Notably, although adoptive T_reg_ therapy has reached Phase I/II trials in type 1 diabetes [43], no large trials have specifically addressed T_regs_ in the context of coinfections.

## Supplementary Information

Supplementary Material 1

## Data Availability

The flow cytometry datasets generated and analysed during this study are not publicly archived to safeguard participant confidentiality, but they are available from the corresponding author on reasonable request.

## Abbreviations

ATRH: Asella Teaching and Referral Hospital
cART: Combination antiretroviral therapy
CRP: C-reactive protein
DMSO: Dimethyl sulfoxide
FCS: Fetal calf serum
Foxp3: Forkhead box protein 3
H. pylori: *Helicobacter pylori*
n.s.: Not significant
PBMC: Peripheral blood mononuclear cell
PBS: Phosphate-buffered saline
PLWH: People living with HIV
TGF-β: Transforming growth factor-β
Th: T helper cell
T_reg_: Regulatory T cell
