# Supplementary material for: Effects of Helicobacter pylori infection on T cell activation markers and regulatory T cells in people with and without HIV infection in Central Ethiopia
Source: Eur J Med Res. 2026 Feb 11;31:349. doi: 10.1186/s40001-026-04025-4 (PMC12930944; doi:10.1186/s40001-026-04025-4)
Supplement: Supplementary file 1 — Supplementary Material 1 [file 40001_2026_4025_MOESM1_ESM.docx]

Supplementary Table 1. Panels for flow cytometry analysis

| **Channel** | **Panel 1**  **(activated T cells)** | **Panel 2**  **(T cell exhaustion)** | **Panel 3**  **(T_regs_)** | **Panel 4**  **(Th17)** |
| --- | --- | --- | --- | --- |
| eFluor 450 | CD4 | CD4 | CD4 | CD4 |
| eFluor 506 | CD3 | CD3 | CD3 | CD3 |
| eFluor 520  (FITC equiv.) | FVD | FVD | FVD | FVD |
| PE | HLA-DR | TIM3 | CD25 | CCR6 |
| PerPE-Cy5.5 | CD8 | CD8 | Foxp3 | CD161 |
| PE-Cy7 | Ki67 | PD-1 | PD-1 | PD-1 |
| eFluor 660 | CD69 | CD57 | CD127 | CD69 |
| APC- eFluor 780 | CD38 | CD38 | CD38 | CD38 |

*_FITC: fluorescein isothiocyanate; PE: phycoerythrin; PerCP: peridinin-chlorophyll-protein complex; Cy: cyanin; APC: allophycocyanin; CD: cluster of differentiation; FVD: fixable viability dye; TIM3: T-cell immunoglobulin and mucin-domain containing-3; Foxp3: forkhead box protein 3; PD-1: programmed death 1_*

# Supplementary Table 2. T cell marker expression by HIV status

| **Marker** | **Subset** | **HIV- Median (95% CI)** | **HIV+ Median (95% CI)** | **Mann–Whitney p** | **Bonferroni p** |
| --- | --- | --- | --- | --- | --- |
| HLA-DR⁺CD38⁺ | CD4⁺ | 1.78%  (1.48–2.03) | 1.76%  (1.41–2.31) | 0.91 | 1.0 |
| Ki67 | CD4⁺ | 88.10%  (84.20–90.20) | 96.10%  (93.90–97.30) | <0.0001 | <0.0001 |
| PD-1 | CD4⁺ | 14.10%  (12.70–15.60) | 19.30%  (16.40–22.00) | <0.001 | <0.01 |
| TIM-3 | CD4⁺ | 1.55%  (1.28–1.98) | 3.43%  (2.76–4.40) | <0.0001 | <0.0001 |
| Th17 (CCR6⁺CD161⁺) | CD4⁺ | 0.30%  (0.20–0.46) | 0.64%  (0.46–1.02) | <0.0001 | <0.001 |
| HLA-DR⁺CD38⁺ | CD8⁺ | 0.49%  (0.38–0.70) | 0.43%  (0.36–0.54) | 0.10 | 0.41 |
| Ki67 | CD8⁺ | 5.56%  (4.17–6.84) | 7.22%  (6.05–10.10) | 0.0010 | 0.0041 |
| PD-1 | CD8⁺ | 0.66%  (0.46–0.95) | 0.85%  (0.75–1.04) | 0.039 | 0.16 |
| TIM-3 | CD8⁺ | 3.45%  (2.82–4.15) | 7.26%  (6.13–9.40) | <0.0001 | <0.0001 |

*_Median percentages (with 95% confidence intervals, CI) of CD4⁺ and CD8⁺ T cell markers in participants with and without HIV. Group differences were evaluated using the Mann–Whitney U test, with Bonferroni correction applied separately for CD4⁺ (5 tests, α=0.01) and CD8⁺ (4 tests, α=0.0125) subsets._*

**Supplementary Figure 1. Frequency of CCR6⁺CD161⁺ CD4 T cells in H. pylori⁻ and H. pylori⁺ individuals stratified by HIV status.** Scatter plots display medians with 95% confidence intervals (CI). Data are shown separately for HIV⁻ (left panel; H. pylori⁻ n = 40, H. pylori⁺ n = 83) and HIV⁺ (right panel; H. pylori^−^ n = 56 and H. pylori^+^ (n = 65)) individuals. Statistical evaluation employed the Mann–Whitney U test; raw p-values are shown in the plots. n.s. denotes non-significant.

**
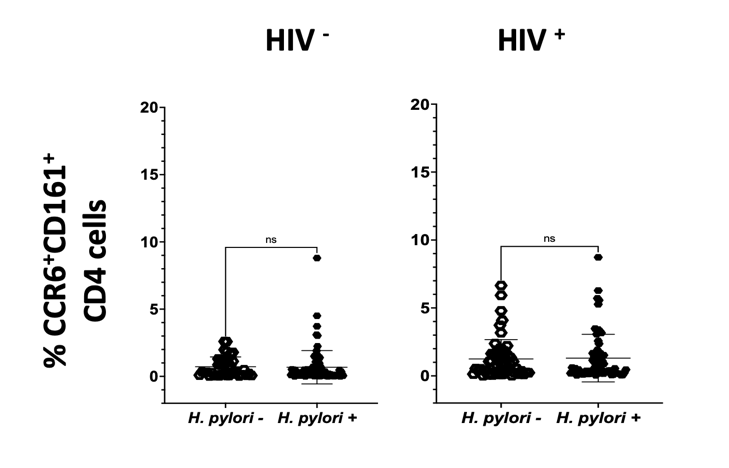
**
